# Population Pharmacokinetics of Caspofungin among Extracorporeal Membrane Oxygenation Patients during the Postoperative Period of Lung Transplantation

**DOI:** 10.1128/AAC.00687-20

**Published:** 2020-10-20

**Authors:** Qianlin Wang, Zhu Zhang, Donglin Liu, Wenqian Chen, Gang Cui, Pengmei Li, Xianglin Zhang, Min Li, Qingyuan Zhan, Chen Wang

**Affiliations:** a Department of Pulmonary and Critical Care Medicine, Center of Respiratory Medicine, China-Japan Friendship Hospital, National Clinical Research Center for Respiratory Diseases, Beijing, China; b Graduate School of Peking Union Medical College, Chinese Academy of Medical Sciences, Peking Union Medical College, Beijing, China; c Department of Pharmacy, China-Japan Friendship Hospital, Beijing, China; d Graduate School of Capital Medical University, Beijing, China

**Keywords:** extracorporeal membrane oxygenation, pharmacokinetics, caspofungin, lung transplantation

## Abstract

Little is known about the influence of extracorporeal membrane oxygenation (ECMO) on the pharmacokinetics (PK) of caspofungin. The aim of this study was to describe population PK of caspofungin in patients with and without ECMO during the postoperative period of lung transplantation (LTx) and to investigate covariates influencing caspofungin PK. We compared ECMO patients with non-ECMO patients, and patients before and after ECMO weaning as self-controls, to analyzed changes in caspofungin PK. Eight serial blood samples were collected from each patient for PK analysis. The population PK of caspofungin was described using nonlinear mixed-effects modeling. Twelve ECMO and 7 non-ECMO lung transplant recipients were enrolled in this study. None of the patients received renal replacement therapy during any part of the study period. The PK of caspofungin was best described by a two-compartment model. There were no significant differences in the PK parameters and concentrations of caspofungin among the ECMO, non-ECMO, and self-control group. In the final covariate model, we found that there was a significant association between the male gender and increased distribution volume, that a higher sequential organ failure assessment score was related to an increase in intercompartmental clearance, and that a longer operative time was related to an increase in clearance and the volume of distribution. ECMO did not have a significant impact on the PK of caspofungin in patients after LTx. Some factors were identified as statistically significant covariates related to the PK of caspofungin; however, their impact on clinical practice of caspofungin needs to be investigated further in more studies. (This study has been registered at ClinicalTrials.gov under identifier NCT03766282.)

## Abstract (precis)

Little is known about the influence of extracorporeal membrane oxygenation (ECMO) on the pharmacokinetics (PK) of caspofungin. The aim of this study was to describe population PK of caspofungin in patients with and without ECMO during the postoperative period of lung transplantation (LTx) and to investigate covariates influencing caspofungin PK. We compared ECMO patients with non-ECMO patients, and patients before and after ECMO weaning as self-controls, to analyzed changes in caspofungin PK. Eight serial blood samples were collected from each patient for PK analysis.

## INTRODUCTION

Fungal infection is associated with high morbidity and mortality rates in patients following lung transplantation (LTx) (1–3). Antifungal prophylaxis in lung transplant recipients may reduce the incidence of fungal infections and the risk of death (4). However, there is no optimized method for the prophylaxis of fungal infection after lung transplantation that is commonly accepted. In our center, we use a combination of intravenous triazoles and inhaled amphotericin B for targeted prophylaxis in patients with a high risk of invasive fungal infections, such as patients with cystic fibrosis and/or fungal colonization of the airways. In patients without high-risk factors, triazole can be replaced by caspofungin for universal antifungal prophylaxis because of its lower toxicity, slight interaction with immunosuppressive drugs, and broad-spectrum activity against most *Candida* species (5), which account for the majority (43 to 80%) of the fungal infections occurring within 1 month of lung transplantation (6, 7).

Extracorporeal membrane oxygenation (ECMO) is by far the most commonly used extracorporeal life support (ECLS) system during the perioperative period of LTx (8, 9). Venoarterial ECMO (VA-ECMO) provides support for both the heart and the lungs, while venovenous ECMO (VV-ECMO) only provides respiratory support. An increasing number of studies have shown that ECMO is associated with significant pharmacokinetic (PK) alterations, including an increased volume of distribution and reduced clearance (10–13). Studies on whether the PK of caspofungin will change in patients receiving ECMO are very limited and controversial. A case report by Spriet et al. reported that the blood concentration of a patient receiving caspofungin during ECMO was maintained at an adequate level (14). However, in another study, Ruiz et al. observed that caspofungin was nondetectable in the ECMO patient receiving the standard dose of caspofungin (15). A change in the PK of caspofungin in ECMO patients inevitably leads to unfavorable levels of prophylaxis with regard to fungal infection. Since our center routinely uses caspofungin for universal antifungal prophylaxis in patients without a high risk of invasive fungal infection, in the present study, we aimed to investigate the PK of caspofungin in patients receiving ECMO after LTx.

## RESULTS

### Demographics and clinical characteristics.

**FIG 1:**
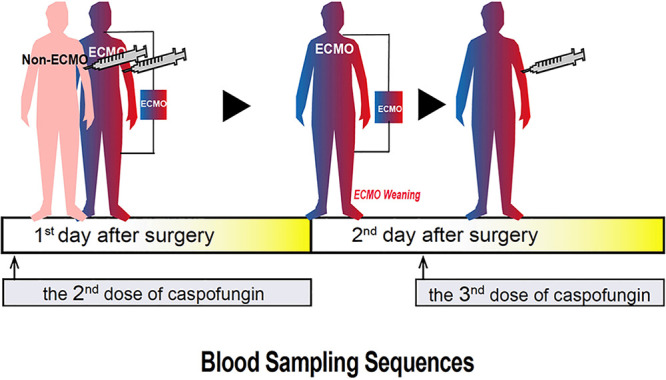
Caspofungin sampling sequences in lung transplant recipients after surgery. The pink person represents the non-ECMO patients, that is, the control group B patients. The blue and red persons with ECMO represent ECMO group patients. The blue and red persons without ECMO represent control group A patients.

All patients were recruited following the acquisition of informed consent. Twelve patients who received ECMO support for more than 24 h and successfully weaned from ECMO were enrolled in the study. We also recruited 7 patients who had never used ECMO support after lung transplantation. Samples were collected from patients in the ECMO group and control group B on the first day after surgery (the 2nd dose of caspofungin). Since all patients in the ECMO group were weaned from ECMO in the morning on the second day after surgery, we sampled patients in control group B at the time when the 3rd dose of caspofungin was given (Fig. 1). Overall, a total of 271 blood samples were collected for PK analysis. The demographic and baseline characteristics of all patients are shown in Table 1, while clinical characteristics are shown in Table 2. There was no significant difference in terms of clinical data between the ECMO group and control group A or between the ECMO group and control group B. It is worth noting that none of the patients experienced hypoalbuminemia during the study and had a negative fluid balance following surgery. All patients in the ECMO group required VV-ECMO support. The median (interquartile range [IQR]) duration of ECMO was 47.8 h (45.9 to 48.8 h). During the study period, the median (IQR) ECMO blood flow rate was 2.9 liters/min (2.3 to 3.0 liters/min).

**TABLE 1:** Demographics and baseline characteristics of study patients^*a*^

Parameter	Value(s) for:		*P* value
	ECMO group (*n* = 12)	Control group B (*n* = 7)	
Age, yr	65 (60–67)	59 (56–62)	0.18
Female, *n* (%)	3 (25.0)	2 (28.6)	0.26
Weight, kg	64 (59–69.3)	65 (53–65)	0.45
BMI, kg/m^2^	22.2 (20.2–24.7)	21.4 (20.5–23.6)	0.97
Double-lung transplantation, *n* (%)	3 (25.0)	6 (85.7)	0.01
Operative time, h	4.0 (3.5–5.0)	5.5 (5.0–5.8)	0.06

a Data are *n* (%) or median. IQR, interquartile range; ECMO, extracorporeal membrane oxygenation; BMI, body mass index.

**TABLE 2:** Clinical characteristics of ECMO group and control groups

Parameter^*a*^	Value(s) for^*b*^:			*P*_1_ value^*c*^	*P*_2_ value^*c*^
	ECMO group (*n* = 12)	Control group A (*n* = 12)	Control group B (*n* = 7)		
ALT, U/liter	17.0 (14.8–21.0)	13.5 (9.5–24.0)	20 (16–24.5)	0.27	0.47
AST, U/liter	31.0 (21.0–38.0)	20.0 (18.5–29.8)	46 (37.5–50)	0.08	0.09
ALB, g/dl	40.0 (37.3–41.3)	42.5 (40.3–43.8)	41 (40–46.5)	0.51	0.11
TBIL, umol/liter	13.1 (7.3–15.9)	11.1 (8.5–12.7)	16.0 (10.2–18.3)	0.32	0.40
CL_CR_, ml/min	93.0 (77.6–124.2)	85.2 (69.2–107.0)	81.7 (72.7–91.2)	0.09	0.07
WBC, 10^9^/liter	16.58 (14.1–18.9)	14.8 (13.2–19.7)	16.1 (15.0–20.6)	0.89	0.56
PLT, 10^9^/liter	128.5 (102.3–171.0)	140.0 (89.3–155.5)	160 (121.5–198.0)	0.56	0.40
Hgb, g/liter	101.5 (96.8–107.5)	96.5 (94–102.3)	128.0 (102.5–47.5)	0.14	0.09
PCT, μg/liter	3.04 (0.7–8.0)	2.6 (0.4–12.4)	5.6 (4.8–22.8)	0.93	0.24
Lac, mmol/liter	1.8 (1.5–2.6)	2.1 (1.4–2.6)	2.4 (2.2–2.7)	0.56	0.41
SOFA score	7 (6–10)	6 (5–9)	7 (6–8)	0.07	0.73
24-h fluid balance, ml	−1503 (−1899, −1119)	−1629 (−2059, −1338)	−2003 (−2319, −1653)	0.90	0.07

a ALT, alanine aminotransferase; AST, aspartate aminotransferase; ALB, serum albumin; TBIL, total bilirubin in serum; CrCL, creatinine clearance; WBC, white blood cell; PLT, blood platelet; Hgb, hemoglobin; PCT, procalcitonin; Lac, blood lactic acid; SOFA, sequential organ failure assessment.

b Data are shown with *n* (%) or median (IQR, interquartile range) for each parameter, unless otherwise indicated.

c *P*_1_ value was calculated between the ECMO group and control group A. *P*_2_ value was calculated between the ECMO group and control group B.

### Pharmacokinetic parameters of caspofungin.

**FIG 2:**
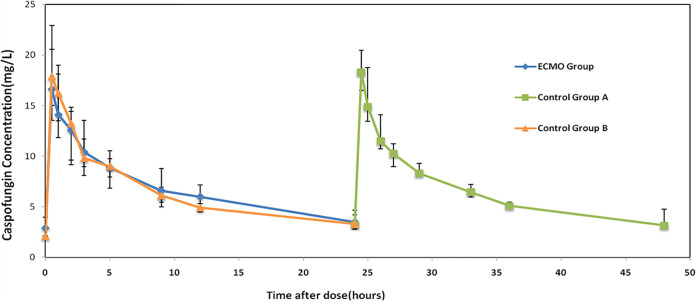
Median concentration-time curves over the 24-h dosing interval in patients with and without ECMO support.

The median (IQR) volume of distribution in the central compartment (*V_c_*) and the volume of distribution in the peripheral compartment (*V_p_*) in the ECMO group was 3.22 liters (2.56 to 3.78 liters) and 2.97 liters (2.03 to 3.50 liters), respectively. Control group A had a *V_c_* of 2.92 liters (2.66 to 3.12 liters) and a *V_p_* of 2.91 liters (2.60 to 3.48 liters), and control group B had a *V_c_* of 3.00 liters (2.49 to 3.35 liters) and a *V_p_* of 2.57 liters (2.27 to 2.58 liters). The clearance rates in the ECMO group, control group A, and control group B were 0.27 liters/h (0.2 to 0.4 liters/h), 0.31 liters/h (0.27 to 0.35 liters/h), and 0.31 liters/h (0.31 to 0.36 liters/h), respectively. There were no statistically significant differences when these pharmacokinetic parameters were compared between groups (Table 3). The median concentration-time curves over the 24-h dosing interval in patients from different groups are shown in Fig. 2; there were no significant differences between the three groups. For all patients, the trough concentration was higher than 1 mg/liter. None of the patients included in this study used drugs that are known to affect the concentration of caspofungin (16), including cyclosporine, rifampin, tacrolimus, efavirenz, nevirapine, phenytoin, dexamethasone, or carbamazepine.

**TABLE 3:** Caspofungin plasma concentrations and pharmacokinetics in the ECMO group and control groups

Parameter^*a*^	Value(s) for^*b*^:			*P*_1_ value^*c*^	*P*_2_ value^*c*^
	ECMO group (*n* = 12)	Control group A (*n* = 12)	Control group B (*n* = 7)		
*C*_max_, mg/liter	16.7 (13.6–20.6)	18.3 (16.5–20.5)	17.9 (15.0–22.9)	0.63	0.64
*C*_min_, mg/liter	3.5 (2.9–4.7)	3.2 (2.9–4.8)	3.5 (3.0–3.6)	0.97	0.89
*V_c_*, liter	3.22 (2.56–3.78)	2.92 (2.66–3.12)	3.00 (2.49–3.35)	0.60	0.19
*V_p_*, liter	2.97 (2.03–3.50)	2.91 (2.60–3.48)	2.57 (2.27–2.58)	0.53	0.35
CL, liter/h	0.27 (0.2–0.4)	0.31 (0.27–0.35)	0.31 (0.31–0.36)	0.58	0.58
AUC_24–48_ (mg·h/liter)	163.06 (121.7–199.2)	147.11 (141.34–174.69)^*d*^	156.2 (143.9–156.7)	0.73	0.74
*t*_1/2_, α, h	1.05 (0.70–1.64)	0.94 (0.91–1.00)	1.09 (0.7–1.14)	0.71	0.87
*t*_1/2_, β, h	16.97 (13.70–20.14)	15.44 (14.09–17.85)	14.25 (12.82–15.38)	0.51	0.08

a *C*_max_, peak plasma concentration; *C*_min_, through plasma concentration; *V_c_*, volume of distribution of the central compartment; *V_p_*, volume of distribution of the peripheral compartment; CL, clearance; AUC, area under the concentration-time curve.

b Data are shown with median (IQR, interquartile range) for each parameter, unless otherwise indicated.

c *P*_1_ value was calculated between the ECMO group and control group A. *P*_2_ value was calculated between the ECMO group and control group B.

d Values are for AUC_48–72_(mg·h/liter).

### Establishment of a pharmacokinetic model.

Based on the concentration-time course for all patients, we established a two-compartment model using NONMEM software. Exponential and proportional error models were used to describe the intersubject and residual variability, respectively. There was no multicollinearity among the selected covariates in the stepwise process. During the process of forward selection, two covariates (operative time and sex) were identified as significant covariates for CL, resulting in a drop in objective function value (OFV) of 11.837 and 5.111 points, respectively. Operative time and sex were also identified as a significant covariate of *V_c_*, resulting in a drop in OFV of 13.492 points and 6.072, respectively. The sequential organ failure assessment (SOFA) score was identified as a significant covariate of intercompartmental clearance (*Q*), resulting in a drop in OFV by 12.354 points. Following backwards elimination, sex for CL was removed from the model (ΔOFV = 3.496). Thus, the final model can be represented by the following equations:

$$\text{CL}={\text{CL}}_{\text{TV}}\times {\left(\frac{\text{OPT}}{5}\right)}^{{\text{θ}}_{{\text{OPT}}_{\text{CL}}}}\times e^{{\text{η}}_{\text{CL}}}$$

$$V_{c}=(Vc_{\text{TV}}+\text{SEX}\times {\text{θ}}_{{\text{SEX}}_{\text{Vc}}})\times {\left(\frac{\text{OPT}}{5}\right)}^{{\text{θ}}_{{\text{OPT}}_{\text{Vc}}}}\times e^{{\text{η}}_{\text{Vc}}}$$

$$Q=Q_{\text{TV}}\times {\left(\frac{\text{SOFA}}{7}\right)}^{{\text{θ}}_{{\text{SOFA}}_{\text{Q}}}}\times e^{{\text{η}}_{Q}}$$

where CL_TV_ is the typical value of total caspofungin CL, *Q*_TV_ is the typical value of caspofungin, *Q*, *Vc*_TV_ is the typical value of caspofungin central volume, and OPT is operative time. Estimates, relative standard errors (RSE), and median parameter estimates (with 95% confidence intervals [CIs]) from 1,000 bootstrap replications are listed in Table 4.

**TABLE 4:** Population PK parameters of final model

Parameter^*a*^	Value by 2-compartment model		Bootstrap value	
	Estimate	RSE (%)	Median	95% CI
Fixed effects				
CL (liter/h)	0.21	8.0	0.26	0.23–0.29
*V_c_* (liter)	2.21	5.0	2.61	2.45–2.89
*V_p_* (liter)	2.87	16.0	2.98	2.30–4.18
*Q* (liter/h)	0.84	11.0	1.00	0.86–1.28
Θ_OPT on CL_	1.30	15.0	1.31	0.84–1.56
Θ_SEX on VC_	0.62	23.0	0.64	0.27–0.93
Θ_OPT on VC_	0.93	14.0	0.87	0.59–1.23
Θ_SOFA on Q_	1.98	21.0	2.04	1.48–2.66
Random effects				
Interindividual variability				
CL (liter/h)	0.04	21.3	0.04	0.01–0.07
*V_c_* (liter)	0.01	15.5	0.01	0.00–0.02
*V_p_* (liter)	0.23	21.2	0.23	0.09–0.45
Residual error				
Additive (mg/liter)	0.73		0.73	0.45–1.08

a *V_c_*, volume of distribution of the central compartment; *V_p_*, volume of distribution of the peripheral compartment; CL, clearance; *Q*, intercompartmental clearance; OPT, operative time; SOFA, sequential organ failure assessment.

**FIG 3:**
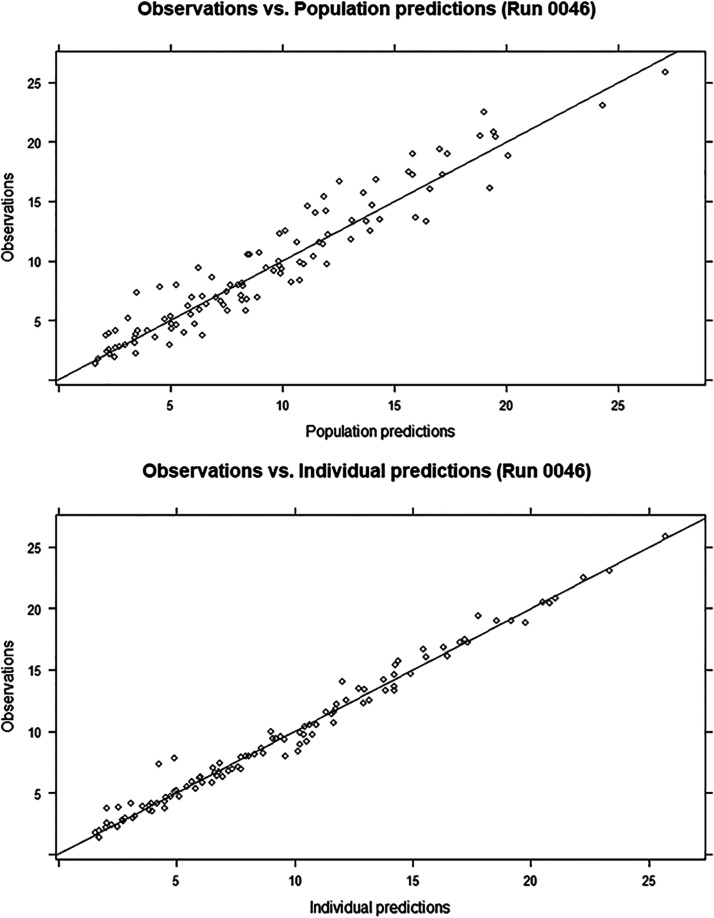
Goodness-of-fit plots for final covariate caspofungin model. The top panel presents the population-predicted concentrations versus the observed concentrations. The bottom panel shows the individual predicted concentrations versus the observed concentrations.

**FIG 4:**
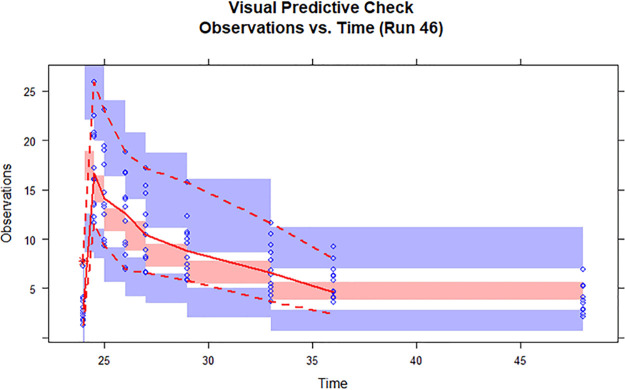
Visual predictive check (VPC) for the final pharmacokinetic model of caspofungin. The open circles represent the observed caspofungin concentrations. The middle solid, lower dashed, and upper dashed lines represent the median, 5th, and 95th percentiles for the observed data, respectively. The shaded areas represent a 95% CI for a simulated predicted median and 5th and 95th percentiles constructed from simulated data sets of individuals from the original data set.

Goodness-of-fit plots created for the PK model showed that there were good correlations between individual predicted concentrations and observed concentrations and between population predicted concentrations and observed concentrations. Furthermore, there was an even distribution of data points on either side of the *y* = *x* line, demonstrating that the data fit the model well (Fig. 3). The final model was validated by visual predictive test (VPC) plots and Bootstrap methods. As shown in the VPC plots (Fig. 4), most of the observed concentration data were included in the 95% prediction interval for the simulated data, indicating that the model has good predictive performance. The parameters of the model fell within the 2.5th to 97.5th percentiles of the Bootstrap parameters, indicating that the established population pharmacokinetic model was stable.

## DISCUSSION

To our knowledge, this is the first prospective population PK study of caspofungin in patients receiving ECMO. Furthermore, this is also the first clinical cohort study of the PK of caspofungin during the postoperative period following LTx. Our analysis showed that ECMO had no significant effect on the PK of caspofungin in patients following LTx.

Case reports have previously shown that ECMO does not affect the PK of caspofungin (14); our present clinical data concur with these earlier data. First, we used the patients as their own control to minimize interindividual variability (IIV). There were no significant differences detected when comparing PK parameters during and after ECMO. Furthermore, there were no significant differences between the ECMO group and control group B. Since patient demographics and clinical data were comparable between the two groups, it was evident that the factors that may affect the PK were minimized. Therefore, ECMO did not appear to have had a significant effect on caspofungin PK. The effect of ECMO on the PK of a drug is mainly due to sequestration in the circuit. Lipophilic drugs, including voriconazole, fentanyl, and propofol, appear to be more significantly sequestered in the ECMO circuit (17–19). However, caspofungin is a freely water-soluble drug and should not, in theory, be sequestered by the ECMO circuit. In a previous study, Spriet et al. (14) reported that adequate levels of caspofungin were maintained in the plasma during ECMO in a critically ill patient, indicating that ECMO did not alter the PK of caspofungin; these findings are consistent with our own. In contrast, an *ex vivo* ECMO study conducted by Shekar et al. showed that the caspofungin recovery rate was only 56% after 24 h of circuit circulation; this may be due to the high protein binding rate of the drug (10). However, *ex vivo* studies have only explored the interaction effect of drugs and circuits and cannot simulate the metabolic processes of drugs in patients, particularly those with severe pathophysiological conditions.

When considering the PK parameters related to caspofungin, we found that the *V* and CL of our LTx patients were significantly lower than those in other critically ill patients (20–26). Several factors may underlie this observed difference. Weight is an important covariate of caspofungin *V* and CL. For example, in a previous publication, Hall et al. (24) showed that as weight increases, the *V* and CL of caspofungin also increase. Weight-based dose regimens have also been proposed for caspofungin (21): 50 mg for patients <70 kg in weight, 70 mg for patients weighing 70 to 100 kg, and 100 mg for patients ≥100 kg in body weight. Accordingly, our present results may have differed from those published previously because our patients had a lower body weight (21–23, 26). Hypoalbuminemia is another factor that can affect both *V* and CL. Previous research found that the *V* of antibiotics with moderate to high protein binding increased by up to 100% in critically ill patients with hypoalbuminemia (27). In patients with normal liver and kidney function, hypoalbuminemia will lead to an increase of free drugs, which can then lead to an increase in CL, particularly in highly protein-bound antibiotics (27), such as caspofungin. Previous research reported that patients experienced various degrees of hypoproteinemia; however, the albumin concentrations were all normal in the patients included in the present study. In addition, as a hydrophilic drug, the *V* of caspofungin can be significantly increased by fluid shifts and large‐volume fluid resuscitation (28); these methods are common in critically ill patients. When hypoalbuminemia occurs, the circulating blood volume is more likely to transfer to the third space, leading to a more significant increase in the *V* of caspofungin (27). However, postoperatively, transplanted lungs experience various degrees of pulmonary edema as a result of increased vascular permeability and severed lymphatic drainage. Therefore, the incorporation of a fluid-restrictive management strategy may be useful in limiting pulmonary edema in such patients (29). This is also the reason for the negative fluid balance of our patients following surgery; this situation differed significantly from the fluid resuscitation treatment for critically ill patients in a state of shock. Therefore, the reasons why *V* and CL were significantly lower in our study than in other studies involving patients who were critically ill may be related to such factors.

In our study, several factors were found to affect the PK of caspofungin. Interestingly, we found, for the first time, that a longer duration of surgery is associated with an increased *V* and CL for caspofungin. The duration of surgery is positively correlated with the dose of anesthetic drugs provided; this may also affect the PK of caspofungin. For example, propofol is one of the drugs used to maintain anesthesia in our study. However, as propofol exhibits high rates of protein binding (98 to 99%) (30), we suspect that this drug competitively inhibited the binding of caspofungin and that this is the main reason for the significant change in the PK of caspofungin (31). In addition, we found that male gender was associated with increased caspofungin clearance. The PK of a drug is affected by multiple body composition parameters; thus, sex differences in body composition parameters may influence the PK of caspofungin (32). Although the screening results for this series of covariates were statistically significant and explain some of the interindividual differences observed, their clinical significance still needs further investigation in more studies.

Our study has several limitations that need to be considered. First, the half-lives of caspofungin that we calculated in our study may not be accurate, because samples were not collected in a steady state. Second, although our study has a limited sample size, it is similar to previous studies concerning the population PK of ECMO patients. Nevertheless, our model can accurately predict the concentration of caspofungin in both individuals and populations. In addition, to minimize interindividual variability and better explore the effects of ECMO on caspofungin, we excluded patients who required renal replacement therapy; this may have affected the generalizability of our current findings. Therefore, it is now necessary to perform larger clinical trials that include a variety of lung transplantation patients.

### Conclusions.

This study serves as an initial step toward understanding the caspofungin PK in lung transplantation patients receiving ECMO and indicates that ECMO has no significant effect on caspofungin PK in these patients. According to the final model, sex, SOFA score, and operative time are the most significant factors influencing the PK of caspofungin.

## MATERIALS AND METHODS

### Design and study population.

This was a prospective, single-center, and open-label pharmacokinetic study. We recruited patients who underwent lung transplantation at the China-Japan Friendship Hospital between October 2017 and March 2018. The inclusion criteria for subjects were the following: (i) patients ≥18 years old; (ii) patients who received ECMO support for more than 24 h postoperatively; and (iii) patients who received caspofungin during their ECMO therapy. We excluded patients with a history of allergy or contraindications to caspofungin, those who required renal replacement therapy, those who were pregnant, and those who had received caspofungin treatment within the 48 h immediately prior to admission. Informed consent was obtained from either the patient or their nominated substitute decision maker. Ethics approval was obtained from the China-Japan Friendship Hospital Ethics Committee in Beijing, China (no. 2018-162-K118-1).

### Control group setting.

This study involved two control groups. Control group A was a self-control group. If a patient in the ECMO group could be weaned from ECMO, then collection of samples for PK analysis was repeated on the first dose in the patient after weaning ECMO. If the patient weaned off ECMO in the morning, caspofungin was given immediately after weaning and then samples were collected. Otherwise, samples would be collected the next morning. Control group B featured patients who had never received ECMO support after lung transplantation and were recruited under the same exclusion criteria. The samples for PK analysis in control group B were collected at the time of the first dose of caspofungin after surgery.

### ECMO apparatus and data collection.

The mode and settings of ECMO were determined based on the clinical context. The ECMO circuit consisted of polyvinyl chloride tubing, a polymethyl-pentene membrane oxygenator (BE-PLS 2050; MAQUET, Hechingen, Germany), a blood pump (BE-PLS 2050; MAQUET, Hechingen, Germany), and a heat exchanger. The ECMO circuit was primed with 600 ml of normal saline. For each patient, we recorded a range of demographic and clinical data, including age, sex, weight, height, body mass index (BMI), mode of ECMO, and operative time. We also recorded laboratory findings, 24-h fluid balance, sequential organ failure assessment (SOFA) score, and ECMO settings on the sampling day.

### Drug regimen and the collection of pharmacokinetic data.

Caspofungin (Cancidas; MSD Sharp & Dohme, Haar, Germany) was administered by intravenous infusion at a dose of 50 mg every 24 h without a loading dose. The first dose of caspofungin was given immediately after surgery in all patients, and we started to sample patients in the ECMO group and control group B at the 2nd dose of caspofungin from the first day after LTx. The sampling dose of patients in control group A depends on the clinical context. Blood samples (1 ml) were obtained from indwelling arterial lines just before the administration of caspofungin and 0.5, 1, 2, 4, 8, 12, and 24 h after the start of the infusion. All blood samples were centrifuged at 3,000 rpm for 5 min, and then supernatant plasma samples were stored at −80°C immediately. Further analysis was carried by the Department of Pharmacy at the China-Japan Friendship Hospital, Beijing, China.

### Drug assay.

The concentration of caspofungin was determined by ultraperformance liquid chromatography-tandem mass spectrometry (UPLC-MS/MS). Caspofungin acetate-d4 was used as an internal standard. A Waters BEH C_18_ column (50 by 2.1 mm, 1.8 μm) was used with a gradient elution with mobile phase A (water with 0.1% formic acid and 2 mmol/liter of ammonium acetate) and mobile phase B (acetonitrile containing 0.1% formic acid). The dynamic range of the assay for caspofungin was 0.39 to 50 mg/liter. Intra- and interday accuracy was ranged from 95.6% to 102.7%. Intraday precision varied between 0.5% and 8.8%, and interday precision varied between 8.6% and 12.7%.

### Development of basic structure model.

Population PK data were processed by the nonlinear mixed effects modeling software NONMEM 7.2 (Icon Development Solutions). We tested one-, two-, and three-compartment pharmacokinetic models to fit the data and selected the most suitable basic structural model according to the −2 log unit likelihood of the objective function value (OFV) and by visually inspecting diagnostic plots.

### Establishment of a random-effect model.

The random-effect model included interindividual variability (IIV) and residual error (RE). The residual error model was used to describe residual variability of the population PK model and was fitted by a combined model with both proportional error and additive errors, according to the following equation:

$$C_{\text{obs}}=C_{\text{ipred}}\left(1+\varepsilon_{1}\right)+\varepsilon_{2}$$

where *C*_obs_ represents the observed plasma concentration and *C*_ipred_ represents the individual predicted concentration. ε_1_ and ε_2_ were the random residual effects for a concentration with a mean of 0 and a variance of σ_1_^2^ and σ_2_^2^, respectively.

Individual variables were then fitted to an exponential error model, according to the following equation:

$$P_{\text{i}}=\text{TV }\left(P\right)\times \text{EXP }\left(\eta_{i}\right)$$

Where *P_i_* represents the individual parameter value for the *i*th patient. TV (*P*) represents the typical individual parameter value, and η*_i_* represents a random variable that is normally distributed with a mean equal to 0 and a variance equal to ω^2^.

### Establishment of a covariate model.

Covariates were considered for inclusion in the final model if they were biologically plausible and showed statistical significance in the base model. In other words, the covariates showed a reduction of OFV by at least 3.84 U (χ^2^ distribution, degrees of freedom [df] = 1, *P* < 0.05) and/or an improvement of goodness-of-fit plots. The following potential demographic or physiological covariates were investigated with regard to their potential influence on the PK parameters of caspofungin: sex, age, weight, BMI, alanine aminotransferase (ALT), aspartate aminotransferase (AST), albumin (ALB), total bilirubin (TBIL), creatinine clearance (CL_CR_), procalcitonin, 24-h fluid balance, presence of ECMO, SOFA score, and operative time. Prior to testing, we diagnosed multicollinearity among the selected covariates by following a stepwise covariate methodology. For continuous covariates, the influence of the covariate (e.g., for caspofungin, CL) was modeled as $\text{CL}i={\text{CL}}_{\text{TV}}\times {\left(\frac{{\text{Cov}}_{i}}{{\text{Cov}}_{m}}\right)}^{\theta_{{\text{Cov}}_{\text{CL}}}}\times e^{\eta_{\text{CL}i}}$, where Cov*_i_* represents the value for subject *i*, Cov*_m_* represents the median value of the covariates, and Cov_CL_ represents the fixed effect of the covariate on CL. For categorical covariates, the influence of the covariate on CL was modeled as $\text{CL}i={\text{CL}}_{\text{TV}}\times e^{\theta_{{\text{Cov}}_{\text{CL}}}}\times e^{\eta_{\text{CL}i}}$. Covariate model building was then performed in a stepwise fashion with forward inclusion and backward deletion. The best model was defined as the one in which the fitting value generated by the model was the closest to the corresponding observation value, that is, the minimum value of the OFV. A change in the OFV was caused by covariate inclusions, and a reduction in OFV to >3.84 (χ^2^, df = 1, *P* < 0.05) was considered the standard for including covariates in the basic model and vice versa. After establishing the full regression model, the final model was obtained by deleting the covariates of each parameter one by one. A recursive backwards elimination procedure was then performed to further refine the model, and a covariate would be removed from the model if the increase in OFV was less than 6.64 (χ^2^, df = 1, *P* < 0.01) during the exclusion.

### Model validation.

The goodness of fit of the model was evaluated by considering two diagnostic plots: the individual predicted concentrations versus the observed concentrations and the population predicted concentrations versus the observed concentrations. The stability and predictive performance of the model were tested by a nonparametric bootstrap method (*n* = 1,000) and a visual predictive test (VPC).

### Statistical analysis.

Statistical analysis was performed using SPSS version 17.0 (SPSS Inc., Chicago, IL). Categorical data are presented as counts (percentages), and continuous data are presented as means ± standard deviations (SD) or medians (IQR). Demographics and clinical differences between study groups were assessed using chi-squared test, Fisher’s exact test, and *t* test, as appropriate. A *P* value of <0.05 was considered statistically significant.

### Ethics approval and consent to participate.

Ethics approval was obtained from the China-Japan Friendship Hospital Ethics Committee (no. 2018-162-K118-1), Beijing, China.

Written informed consent was received from the patients or their legal representatives.

### Availability of data and material.

The datasets used and/or analyzed during the current study are available from the corresponding author on reasonable request.
